# Internal Transmesenteric Hernia Causing Small Bowel Obstruction: A Case Report

**DOI:** 10.7759/cureus.86498

**Published:** 2025-06-21

**Authors:** Pragya Sinha, Virendra S Chauhan, Surabhi Gupta

**Affiliations:** 1 Radiodiagnosis, Indraprastha Apollo Hospitals, New Delhi, IND; 2 Radiodiagnosis, Saral Diagnostics, Noida, IND

**Keywords:** abdominal emergency, diagnosis of mesenteric internal hernia, internal abdominal hernia, mesenteric hernia, mesenteric internal hernia

## Abstract

Internal hernias often present with vague and nonspecific symptoms of bowel obstruction. However, they carry a potential for bowel ischemia; as such, they need immediate surgery. It is important to identify the etiology of small bowel obstruction as it may cause ischemia or strangulation of the bowel. Most guidelines suggest a wait-and-watch policy for small bowel obstruction. At the same time, if there are any signs of peritonitis, strangulation, or bowel ischemia, surgical exploration is recommended.

We present a case of an 81-year-old man who came to the emergency department with complaints of vague but persistent abdominal pain. The pain was localized to the right flank and had become dull in nature. An abdominal X-ray showed a few air-fluid levels suggestive of subacute obstruction. The portable ultrasound was inconclusive. A non-contrast computed tomography (NCCT) scan of the abdomen was done for the non-resolution of pain. It revealed rotation/volvulus in the small bowel mesentery. A deeper look suggested the possibility of internal transmesenteric herniation with evidence of early bowel ischemia. Surgical exploration revealed ileal loops internally herniating inside a band of mesenteric tissue. The mesenteric band was cut, and the obstruction was relieved. Subsequently, the patient was discharged home.

## Introduction

Small bowel obstruction is a common cause of abdominal pain in patients presenting to the emergency department. In the United States, 65-75% of small bowel obstruction cases are due to post-surgical adhesions [1,2]. After post-surgical adhesions, its most common causes in developed countries are hernias (10%) and neoplasms (5%), respectively [1]. Intussusception, inflammatory bowel disease, gallstone ileus, intestinal volvulus, internal hernia, foreign bodies, and bezoars are the rare causes of obstruction. Across the world, hernias, both external and internal, form the most common cause of small bowel obstruction [2-4]. Internal hernias account for less than 1% of all intestinal obstruction cases (incidence rate: 0.2-0.9%) [1].

The Bologna guidelines on acute small bowel obstruction suggest a trial of non-operative management in all patients [5]. However, in a certain subset of cases, particularly if there are signs of peritonitis, strangulation, or bowel ischemia, immediate operative management is needed. All-cause mortality for small bowel obstruction may increase up to 30% if any complication, like bowel ischemia, has occurred [1].

An internal hernia is the herniation of a bowel loop through a mesenteric window or into a peritoneal sac. Internal hernias of the small bowel are a relatively rare cause of small bowel obstruction. They account for less than 1% of all intestinal obstructions and ~6% of all small bowel obstructions [6]. However, they should be accurately diagnosed early due to the slightly higher [6] potential for bowel ischemia, strangulation, and mortality if left untreated. Any case of non-resolving bowel obstruction needs to be further investigated with a non-contrast computed tomography (NCCT) scan of the abdomen. 

We present the case of an 81-year-old man who came to the emergency department with non-resolving dull pain in the abdomen. Further investigation showed bowel obstruction likely caused by an internal mesenteric herniation of the small bowel. Immediate operative management relieved the band of tissue causing the internal hernia.

## Case presentation

The patient, an 81-year-old man, came to the emergency department with complaints of dull right-sided abdominal pain. At the time of onset, ~8 hours ago, pain was sharp and severe in nature. The subject was associating it with the recently administered shingles vaccine. However, no evidence for the same was present. 

**Figure 1:**
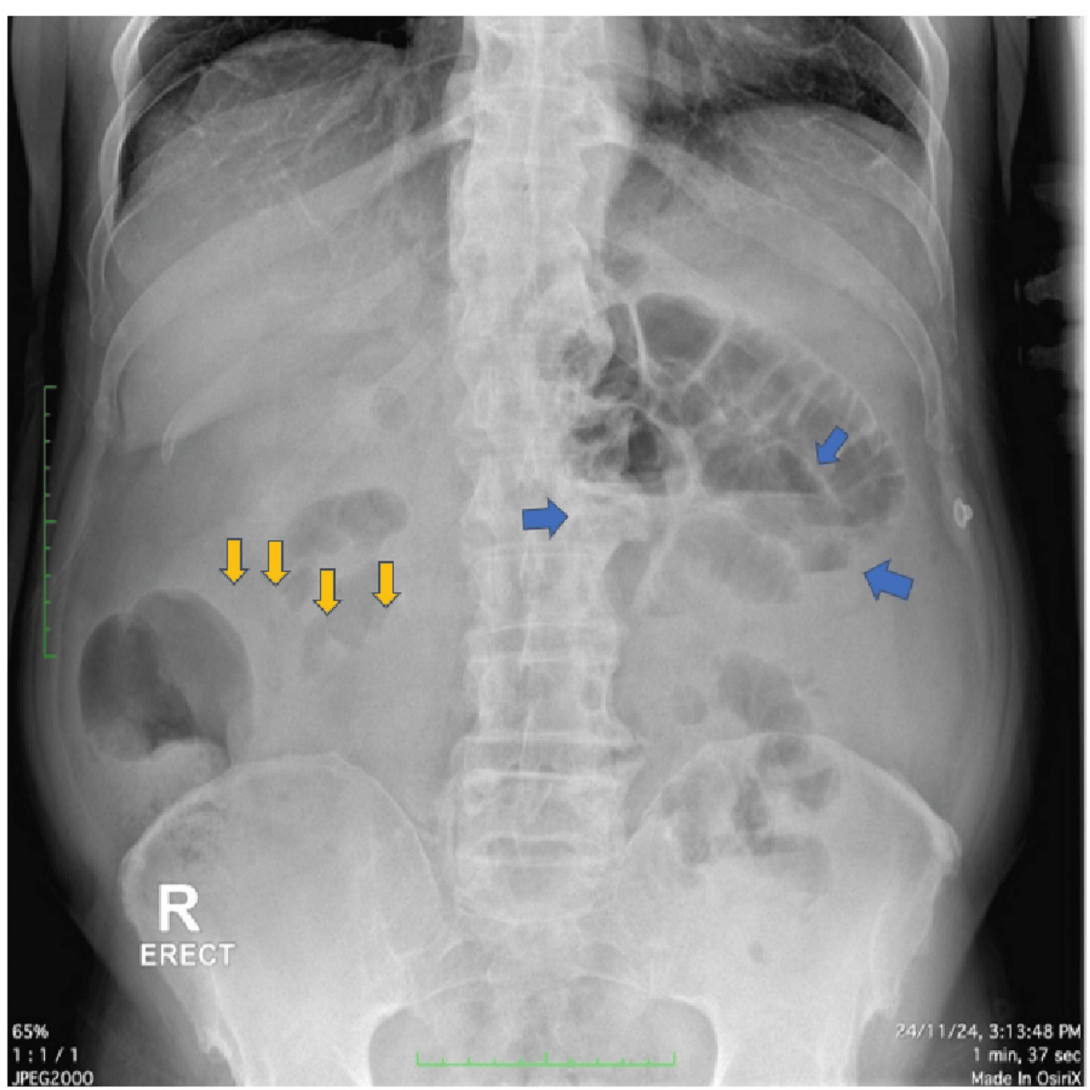
X-ray of the abdomen showing a gasless abdomen (yellow arrows) with few air-fluid levels (blue arrows) suggesting some degree of bowel obstruction

Initial portable ultrasound was inconclusive with the presence of fatty liver, mild prostatomegaly, acute urinary retention, and features of cystitis. However, the abdominal X-ray showed a gasless abdomen with few air-fluid levels in the left hypochondrium, suggesting some degree of bowel obstruction (Figure 1). The patient had no prior history of abdominal surgery. However, he was a known case of chronic kidney disease with a creatinine known to be in the range of 1.2-1.6 mg/dL (normal: <1 mg/dL). In view of the patient's age, an electrolyte sample was sent to rule out paralytic ileus. The electrolyte panel came back normal. 

**Figure 2:**
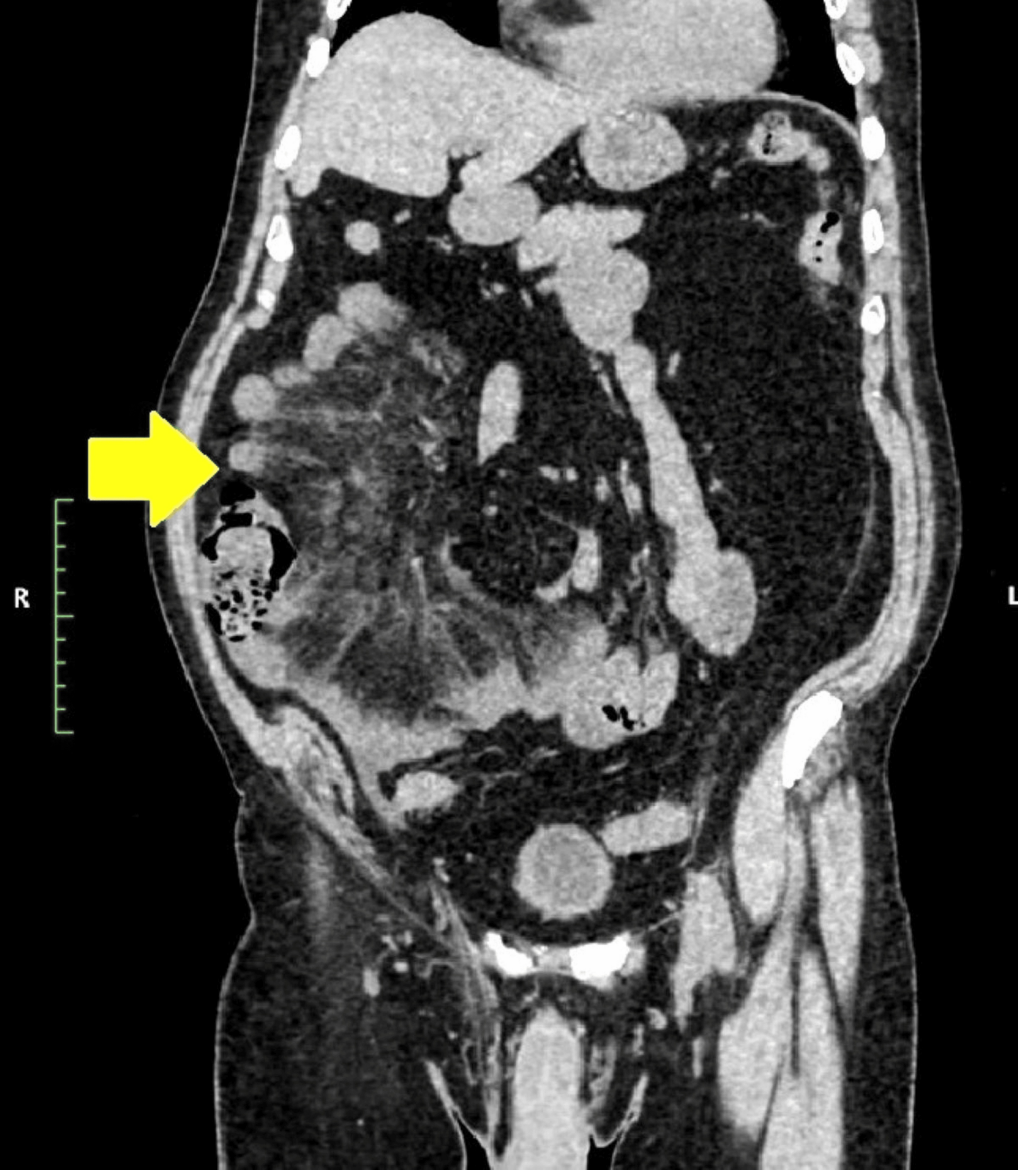
Mesenteric fat stranding in the right lower quadrant with clumping of small bowel loops (yellow arrow) on the right side of the abdomen

**Figure 3:**
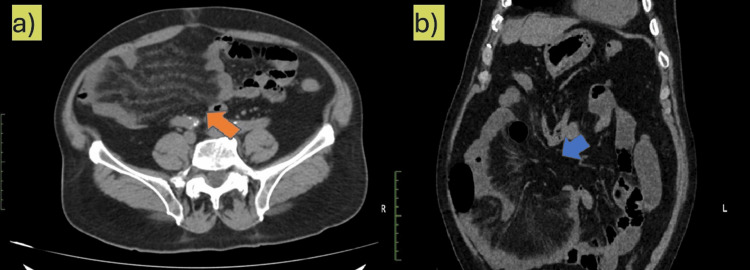
A non-contrast computed tomography scan of the abdomen showing (a) mesenteric fat stranding seen distal to a small area of narrowing (orange arrow) and (b) the area of trapping/looping of the mesentery on the right side of the abdomen distal to a narrowing (blue arrow) suggestive of a mesenteric defect

**Figure 4:**
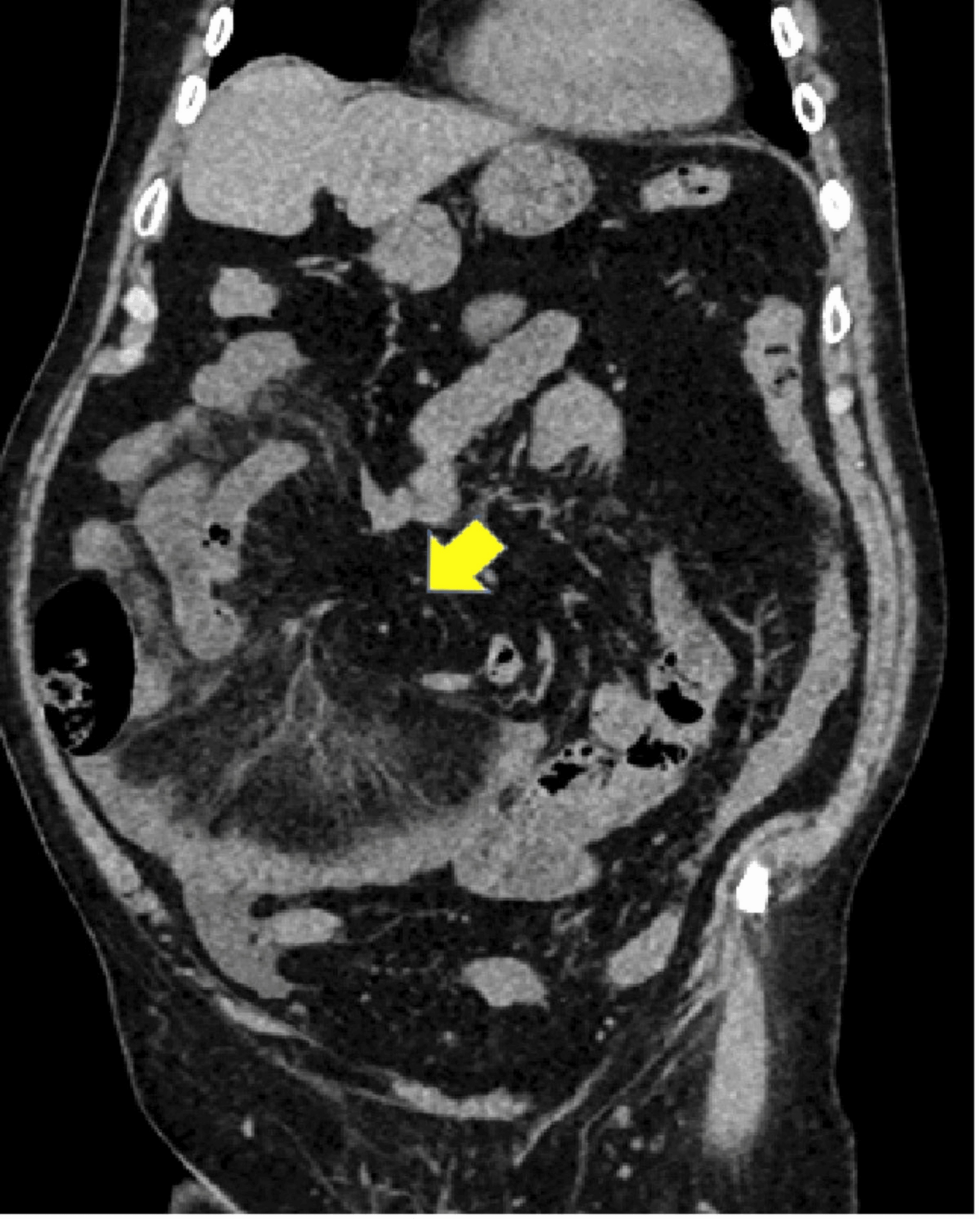
Whirlpool sign of mesenteric rotation seen in a coronal non-contrast computed tomography scan distal to the area of mesenteric narrowing (yellow arrow)

An NCCT scan of the abdomen was done due to the non-resolution of symptoms. NCCT of the abdomen revealed clustering of the small bowel loops in the right lower abdomen (Figures 2-3 and Video 1). Additionally, there was significant mesenteric fat stranding in the right lower abdomen, predominantly involving the ileal mesentery. Closer examination revealed the presence of a whirlpool sign (Figure 4) with rotation of the small bowel mesentery, suggesting some component of small bowel volvulus. A contrast CT scan was suggested on this basis. However, creatinine level was raised (1.6 mg/dL) at the time of CT scan. In view of advanced age and mildly raised creatinine, a contrast scan was not done. A second look at the NCCT showed an area of trapping/looping of the mesentery in the lower lumbar region. Mesenteric stranding was present distal to the small area (Video 2) of bowel trapping. A possibility of internal herniation with consequent mesenteric volvulus was tentatively suggested.

**Video 1:** A non-contrast computed tomography scan on the first day of admission showing clustering of bowel loops in the right lower quadrant along with mesenteric stranding in the same region

**Video 2:** The second non-contrast computed tomography scan section of the abdomen showing mesenteric fat stranding, clustering of bowel loops in the right lower abdomen, and small bowel whirlpool sign suggesting mesenteric rotation

**Figure 5:**
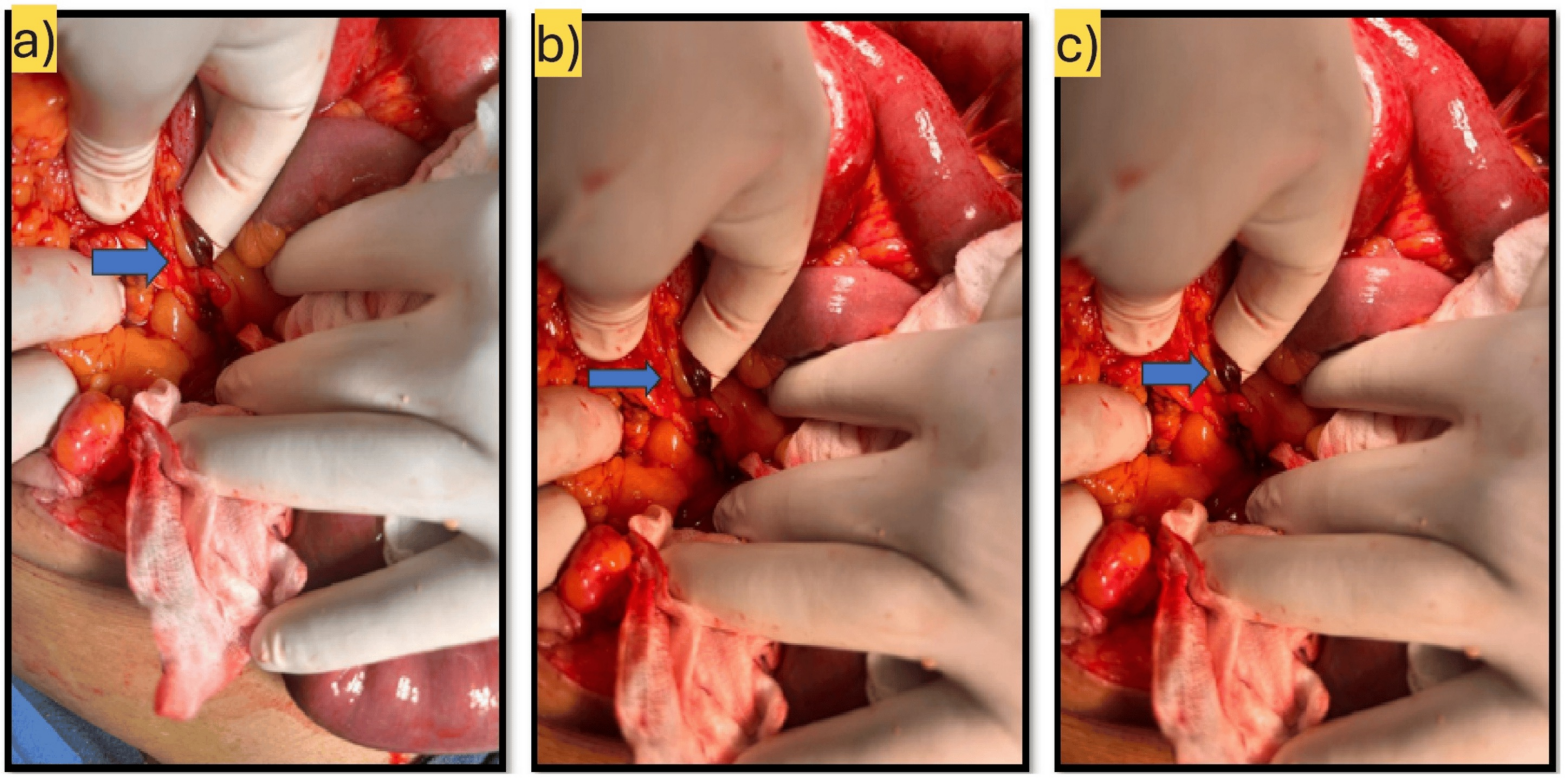
Mesenteric band (blue arrows) causing defect seen during surgery (a-c)

The subject was operated upon. A band of mesenteric tissue was found to be creating a mesenteric defect. This was causing the internal herniation of ileal loops along with the rotation of the mesentery (Figure 5). The mesenteric band was released, and the subject showed resolution of the pain. Postoperatively, the patient had a bout of pneumonia in recovery. After a course of antibiotics, he was discharged home. Further history taking and exploration showed that the patient had previously been admitted in 2012 with a similar complaint (Video 3). The mesenteric defect was likely present from a younger age and was only now discovered due to the bowel strangulation. Since the subject had a previously non-operated "virgin" abdomen, the mesenteric defect was postulated to be post-inflammatory in nature. It went undetected due to a lack of ischemic changes in previous episodes. This case is unique in that the patient has no history of prior surgical history. Furthermore, the raised creatinine and age category were limiting the patient management.

**Video 3:** A non-contrast computed tomography scan of the abdomen from 2012 showing similar clustering and looping without features of intestinal obstruction

## Discussion

Acute bowel obstruction remains a common cause of surgical emergencies. It accounts for ~350,000 emergency admissions every year in the United States [7,8]. A meta-analysis by Yang et al. showed that non-operative management of small bowel obstruction is superior to operative management with a success rate of 96% and a morbidity rate of 3% compared to 89% success rate and 26% morbidity rate in the operative group [9]. This translates into the fact that most cases of small bowel obstruction are left alone since they resolve spontaneously [5,9].

At the same time, certain cases of small bowel obstruction need immediate surgery to save the bowel from strangulation or ischemia. These cases are particularly important to detect radiologically. Bowel ischemia, which may need bowel removal or stoma formation, can cause significant deterioration of the quality of life. Any potentially preventable cause that may lead to bowel ischemia should be detected early. The surgical causes of small bowel obstruction include any type of strangulating/ischemic hernia, volvulus, and tumors of either benign or malignant etiology.

Approximately 6% of all small bowel obstructions are caused by internal herniation [7]. Several classifications exist for internal herniation of the small bowel (Table 1) [4,10,11]. The most widely used classification is by Meyers, who has classified internal hernias based on their location. Paraduodenal hernias are the most common type of internal hernia historically with an incidence of ~53% [4,6,12]. Pericecal hernia, foramen of Winslow hernia, transmesenteric/transmesocolic hernia, intersigmoid hernia, and retroanastomotic hernia are the other types described by Meyers. 

**Table 1:** Different classifications of internal hernias, based on location and type of peritoneal defect

Author	Basis of classification of internal hernias	Categories
Meyers [4,13]	Location	Paraduodenal hernia (53%)
		Pericecal hernia (13%)
		Foramen of Winslow hernia (8%)
		Transmesenteric/transmesocolic hernia (8%)
		Intersigmoid hernia (6%)
		Retroanastomotic hernia (5%)
Ghahremani [10]	Location	Paraduodenal hernias (50-55%)
		Pericecal hernias (10-15%)
		Foramen of Winslow hernias (6-10%)
		Transmesenteric hernias (8-10%)
		Intersigmoid hernias (4-8%)
		Paravesical hernias (<4%)
Lanzetta et al. [14]	Location	1a: Left paraduodenal hernia
		1b: Right paraduodenal hernia
		Foramen of Winslow hernia
		Pericecal hernia
		Sigmoid mesocolon-related hernia
		Transmesenteric hernia
		Transomental hernia
		Paravesical and pelvic hernia
Doishita et al. [11]	Type of peritoneal defect	Herniation through:
		A normal foramen
		An unusual peritoneal fossa or recess into the retroperitoneum
		Abnormal opening in the mesentery or peritoneal ligament

Mesenteric herniation through a defect or abnormal opening in the mesentery remains a relatively uncommon cause of bowel obstruction. The mesentery has the task of providing scaffolding for the peristaltic bowel loops as well as channeling their vascular supply. Therefore, any defect in the mesentery has the potential to cause herniation of the mobile small bowel as well as strangulation and trapping of its vascular supply. Among all types of internal hernias, transmesenteric herniation or herniation through a mesenteric defect has one of the lowest incidences at ~5-10% of all internal hernias [7,15]. Mesenteric hernias can grow to be quite large in the absence of a limiting sac [16]. Additionally, they do not have a fixed location, unlike foramen of Winslow or pericecal hernias. As such, they are difficult to suspect and confirm unless there is a high degree of suspicion for them in all causes of small bowel obstruction [16].

Transmesenteric herniation has a bimodal age distribution. In young children, the mesenteric defect is typically present congenitally. This group is most likely to have internal hernia as a cause of bowel obstruction [15]. In adults, the mesenteric defect usually develops after surgery or prolonged inflammation [17]. Laparoscopic Roux-en-Y gastric bypass done for bariatric surgery has a particular predisposition to cause internal mesenteric herniation [18]. Hence, any abdominal pain in post-gastric bypass surgery is highly suspicious for transmesenteric herniation of the small bowel. The literature has reported very few cases of internal mesenteric herniation in adult subjects with a virgin, non-operated abdomen. Our case is one of the few reports adding to the literature about this relatively rare entity.

Blachar et al. have done the most extensive reporting on transmesenteric hernia. In a review of 14 cases of transmesenteric herniation, they suggested that common NCCT findings include features of small bowel obstruction, such as dilated bowel loops proximal to the site of obstruction with multiple air-fluid levels. There is also clustering of bowel loops in one area of the abdomen, as well as the displacement, engorgement, and crowding of mesenteric vessels. Signs of small bowel volvulus and ischemia are also seen in many of these cases [19]. Additionally, swirling of the mesentery (whirlpool sign) remains the most specific (~80-90%) predictor for internal herniation [18].

## Conclusions

Acute bowel obstruction remains a common cause of surgical emergencies. Unlike large bowel obstruction, most cases of small bowel obstruction are left alone since they resolve spontaneously. At the same time, certain cases of bowel obstruction need immediate surgery to save the bowel from strangulation or ischemia. Bowel infarction, which needs resection or stoma formation, is a preventable cause of significant deterioration in the quality of life. Bowel ischemia can also lead to morbidity and mortality. It is important to identify those cases of obstruction that may progress to ischemia or strangulation as early as possible. The common surgical causes of small bowel obstruction include hernia, volvulus, and tumors of either benign or malignant etiology.

In summary, mesenteric hernias remain an uncommon cause of small bowel obstruction. Due to the absence of a fixed location and the absence of a limiting sac, they can often grow to be very large before detection. Additionally, imaging findings are often nonspecific, which means the final confirmation of etiology is usually surgical. Hence, a high degree of suspicion should be kept when imaging any non-resolving small bowel obstruction. The findings of mesenteric vessel abnormality, swirling of vessels, clustering of bowel loops, and small bowel volvulus should alert the radiologist to search for causes of internal herniation.

## References

[1] Miller G, Boman J, Shrier I, Gordon PH (2000). Etiology of small bowel obstruction. Am J Surg.

[2] Aka AA, Wright JP, DeBeche-Adams T (2021). Small bowel obstruction. Clin Colon Rectal Surg.

[3] Fekadu G, Tolera A, Beyene Bayissa B, Merga BT, Edessa D, Lamessa A (2022). Epidemiology and causes of intestinal obstruction in Ethiopia: a systematic review. SAGE Open Med.

[4] Meyers MA (1983). Dynamic radiology of the retroperitoneum. Normal and pathologic anatomy. Acta Gastroenterol Belg.

[5] Ten Broek RP, Krielen P, Di Saverio S (2018). Bologna guidelines for diagnosis and management of adhesive small bowel obstruction (ASBO): 2017 update of the evidence-based guidelines from the world society of emergency surgery ASBO working group. World J Emerg Surg.

[6] Meyers MA (2005). Internal abdominal hernias. Dynamic Radiology of the Abdomen: Normal and Pathologic Anatomy.

[7] Martin LC, Merkle EM, Thompson WM (2006). Review of internal hernias: radiographic and clinical findings. AJR Am J Roentgenol.

[8] Rami Reddy SR, Cappell MS (2017). A systematic review of the clinical presentation, diagnosis, and treatment of small bowel obstruction. Curr Gastroenterol Rep.

[9] Yang TW, Prabhakaran S, Bell S (2021). Non-operative management for small bowel obstruction in a virgin abdomen: a systematic review. ANZ J Surg.

[10] Ghahremani GG (1984). Internal abdominal hernias. Surg Clin North Am.

[11] Doishita S, Takeshita T, Uchima Y (2016). Internal hernias in the era of multidetector CT: correlation of imaging and surgical findings. Radiographics.

[12] Selçuk D, Kantarci F, Oğüt G, Korman U (2005). Radiological evaluation of internal abdominal hernias. Turk J Gastroenterol.

[13] Nolan D (2003). Dynamic radiology of the abdomen: normal and pathologic anatomy, 5th edn. Gut.

[14] Lanzetta MM, Masserelli A, Addeo G (2019). Internal hernias: a difficult diagnostic challenge. Review of CT signs and clinical findings. Acta Biomed.

[15] Mathieu D, Luciani A (2004). Internal abdominal herniations. AJR Am J Roentgenol.

[16] Blachar A, Federle MP (2002). Internal hernia: an increasingly common cause of small bowel obstruction. Semin Ultrasound CT MR.

[17] Spangler R, Van Pham T, Khoujah D, Martinez JP (2014). Abdominal emergencies in the geriatric patient. Int J Emerg Med.

[18] Lockhart ME, Tessler FN, Canon CL (2007). Internal hernia after gastric bypass: sensitivity and specificity of seven CT signs with surgical correlation and controls. AJR Am J Roentgenol.

[19] Blachar A, Federle MP, Dodson SF (2001). Internal hernia: clinical and imaging findings in 17 patients with emphasis on CT criteria. Radiology.

